# First person – Jorge Rodriguez-Gil

**DOI:** 10.1242/dmm.044222

**Published:** 2020-03-13

## Abstract

First Person is a series of interviews with the first authors of a selection of papers published in Disease Models & Mechanisms (DMM), helping early-career researchers promote themselves alongside their papers. Jorge Rodriguez-Gil is first author on ‘Genetic background modifies phenotypic severity and longevity in a mouse model of Niemann-Pick disease type C1’, published in DMM. Jorge is a postdoctoral fellow in the lab of William J. Pavan (National Human Genome Research Institute) and Frances M. Platt (University of Oxford) investigating the genetic architecture of Niemann-Pick disease type C.


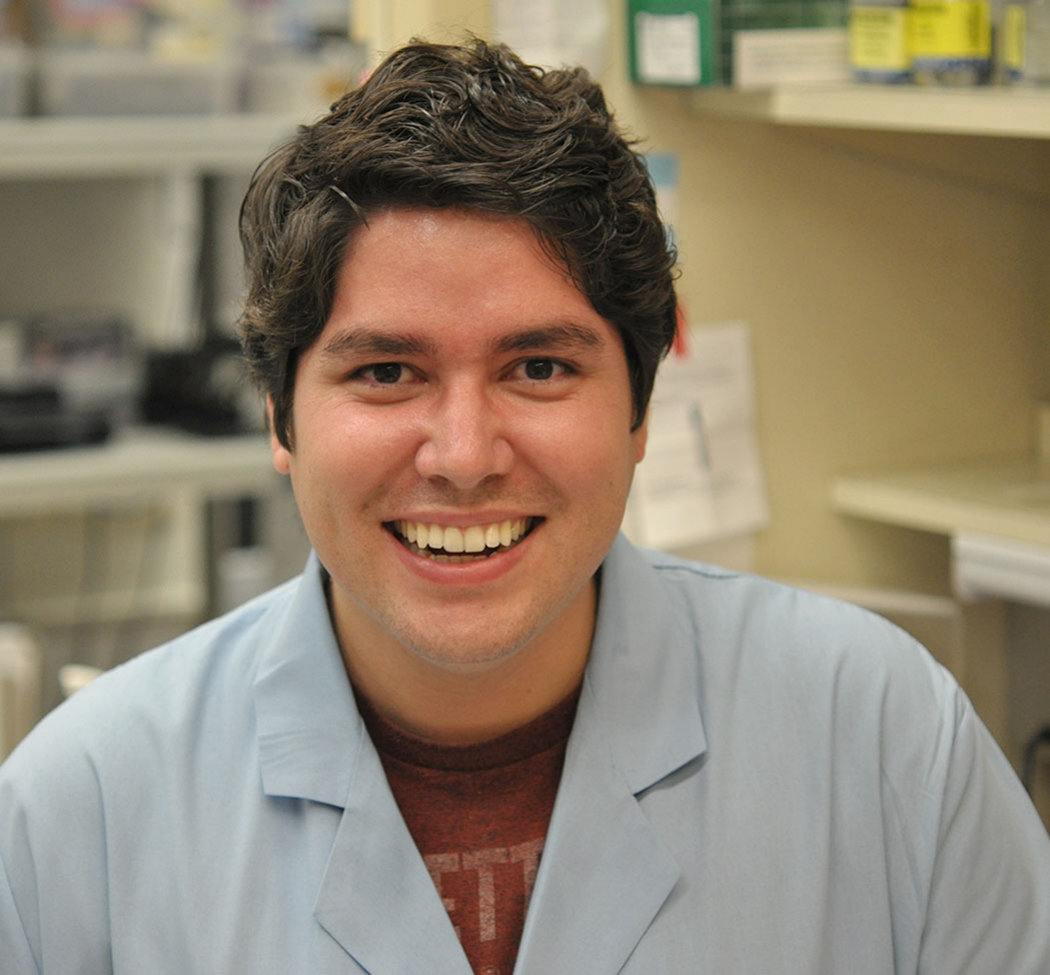


**Jorge Rodriguez-Gil**

**How would you explain the main findings of your paper to non-scientific family and friends?**

Niemann-Pick disease type C1 (NPC1) is a fatal and rare disease in which cholesterol accumulates abnormally inside cells, leading to cell death. This disease is caused by mutations in the *NPC1* gene, which encodes a protein that participates in the transport of different types of lipids within a specialized organelle inside the cells called a lysosome. NPC1 patients can have a wide variety of clinical presentations including an enlarged liver or spleen as well as symptoms affecting their brain. More than 400 disease-causing mutations have been identified in the DNA of NPC1 patients, and even patients with the same mutation can develop very different symptoms. This suggests that other genes besides NPC1, known as genetic modifiers, might be responsible for some of the variation in disease severity. By using an NPC1 mouse model (*Npc1^em^*), we were able to identify regions in the genome in which these potential modifier genes are located.

**What are the potential implications of these results for your field of research?**

The identification of genetic modifiers of NPC1 will have an enormous impact by providing novel insights into mechanisms of disease progression. These candidate genes will also serve as new targets for disease diagnosis and intervention. Furthermore, the discovery of these modifier regions demonstrates that animal models are a powerful tool to study the genetics of rare diseases such as NPC1.

**What are the main advantages and drawbacks of the model system you have used as it relates to the disease you are investigating?**

Animal models are essential to the study of human disease. In rare diseases such as NPC1, model organisms play a particularly vital role, as sample size is often a limiting factor in clinical trial design. In the case of our study, we took advantage of naturally occurring genetic variants specific to different inbred strains of mice to identify modifier regions associated with changes in disease severity and survival. The identification of candidate genes within each modifier region was possible because sequenced genomes were available from different mouse strains. One major drawback of using mice in genetic mapping studies is the need for large numbers, which can result in high costs due to the expense of housing and care compared to other animal models. Also, although the *Npc1^em^* mouse model shows classical phenotypes that are present in human NPC1 patients, there are inherent differences in physiology between mice and humans, and thus results from studies in this mouse model will need to be verified in human cells in the future.

“In rare diseases such as NPC1, model organisms play a particularly vital role, as sample size is often a limiting factor in clinical trial design.”

**What has surprised you the most while conducting your research?**

Because I did not have any animal research experience before I started my PhD, I was very surprised by the high level of similarity in terms of disease phenotype between our mouse model and NPC1 patients.

**Describe what you think is the most significant challenge impacting your research at this time and how will this be addressed over the next 10 years?**

Since the completion of the Human Genome Project, DNA sequence technology has allowed us to identify genes responsible for classical Mendelian disorders such as NPC1 more efficiently than in the past. Further analysis of these mutations has revealed that, even when accounting for their causative effect, phenotypic variation still exists. As a consequence, the term ‘monogenic’ has been called into question for many of these disorders, for which the collection of additional sequence and phenotype data now suggests that genetic modifiers are present. These additional genetic variants will be identified as we continue to use different -omics platforms in conjunction with model organisms.

**Figure:**
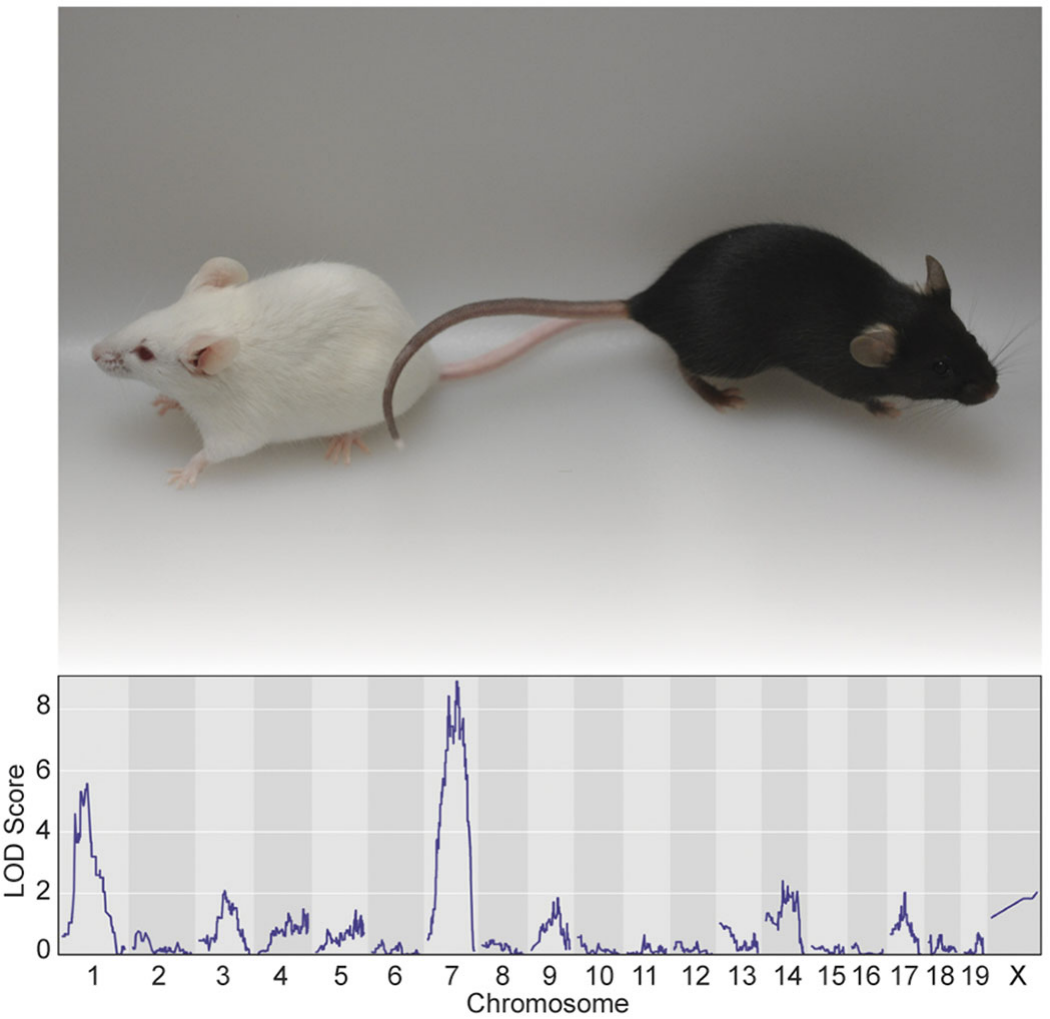
**Strain-specific lifespan differences between *Npc1^em/em^* mutants from two different genetic backgrounds (C57BL/6J and BALB/cJ) map to chromosomes 1 and 7.**

**What changes do you think could improve the professional lives of early-career scientists?**

During their training, I think scientists should be exposed to career pathways that are not necessarily linked to being the head of a lab. Being a good researcher takes critical thinking and creativity – two skills that are highly valuable in many settings, and having trained scientists enter a wide variety of career paths is essential.

“Being a good researcher takes critical thinking and creativity – two skills that are highly valuable in many settings.”

**What's next for you?**

As part of my combined MD/PhD training I will be coming back to finish my last year of medical school. I will also be applying for Paediatric/Medical Genetics residency programmes this fall.
